# Submission policy, peer-review and editorial board members: interesting conflicts and conflicts of interest

**DOI:** 10.1186/1757-7241-18-56

**Published:** 2010-10-26

**Authors:** Kjetil Søreide, Kjetil G Ringdal, Hans Morten Lossius

**Affiliations:** 1 Department of Surgery, Stavanger University Hospital, Stavanger, Norway; 2 Institute of Surgical Sciences, University of Bergen, Bergen, Norway; 3 Department of Research, Norwegian Air Ambulance Foundation, Drøbak, Norway; 4 Institute of Clinical Medicine, Faculty of Medicine, University of Oslo, Oslo, Norway

## 

The *Scandinavian Journal of Trauma, Resuscitation and Emergency Medicine *welcomes an ever-increasing number of submissions while maintaining an acceptance level well below half of all submitted manuscripts,[1] meaning that the number and quality of submitted papers are increasing. As has been stated in the past,[2] the editors endorse a number of guidelines in order to improve presentation and style as well as adherence to current standards in publishing.

Notably, all papers submitted to the SJTREM and potentially deemed suitable for publication will undergo peer-review from at least 2 (and often more) referees before making a final decision to accept or reject. Due to an increasing number of case reports, the decision to immediately reject those deemed unsuitable for the SJTREM has become more rigorous. The SJTREM wishes to maintain a main focus on original articles, review articles and solicited commentaries to selected studies.

While peer-review is currently the best, yet however an imperfect, controversial and, sometimes a misused tool for vesting the scholarly work performed by others[3-5], the editors maintain their right to finally decide whether or not a paper should be published in the SJTREM. Indeed, to paraphrase the recent Lancet editorial by Richard Horton[6] "...*peer review is indispensable ...but we also know that it is widely misunderstood. Peer review is not the absolute or final arbiter of scientific quality...it does not test the validity of a piece of research. It does not guarantee truth. Peer review can improve the quality of a research paper...it tells you something about the acceptability of new findings among fellow scientists*..." where he explores this in a greater context of science[6]. Thus, sometimes the editors will make a decision that may contrast the opinions expressed by the referee(s). In such cases where the editorial decision deviates from that of the referee, the peer-reviewer should still be assured that we pay a great deal of attention to the meticulous work done for the SJTREM. Indeed, we highly appreciate the voluntarily work made by the international group of highly dedicated clinicians and scientists dedicating their time and knowledge to ensure the content of the SJTREM. Knowing that the community of researchers working in the field of trauma, resuscitation and emergency medicine in Scandinavia may not be very large and, indeed, even in the European or international setting may "interesting conflicts" arise where more than one opinion or direction of research is voiced. We truly believe these "conflicts" to be necessary means of furthering opinion and academic progress for the better good of patient care. Thus, we sometimes find it more important to "agree to disagree" than vesting in uniform voices of opinion only.

While editorial integrity is the *sine qua non *for any academic Journal, the editors and editorial board members will, as academically active clinicians and researchers, from time to time submit papers for consideration to "their own" journal, in this case the SJTREM. The readers of SJTREM should rest assured that any means of ensuring that integrity and avoiding a corruptive type of camaraderie has been taken. The responsibility of disclosing any conflicts of interest lies with authors as well as referees and editors. Reviewers, alike with authors and editors, need to declare all conflicts of interest, not only financial ties. Often, competitive issues or personal relationships lead to more important and less obvious biases.

According to the World Association of Medical Editors (WAME), of which SJTREM is also a member, any conflict of interest can be said to exists when "...there is a divergence between an individual's private interests (competing interests) and his or her responsibilities to scientific and publishing activities such that a reasonable observer might wonder if the individual's behaviour or judgment was motivated by considerations of his or her competing interests..."[7]. Obviously, conflicts of interest of some sort will often exist, and some conflicts of interest are unavoidable. Having a conflict of interest is not in itself unethical, and having a competing interest does not, in itself, imply wrongdoing. However, it constitutes a problem when competing interests could influence one's responsibilities in the publication process. As a consequence, research institutions, professional societies, and an increasing number of journals have formulated guidelines for dealing with potential conflicts of interest. Essentially, most of these guidelines require authors to disclose such conflicts either in the cover letter to the editor of the journal and/or in a in the manuscript itself. The Editors of SJTREM recommends authors and referees to readily disclose any potential conflicts in the correspondence with he editors. As Editors, we will likewise seek to avoid situations in which conflicts of interests may be overriding the decision process, yet acknowledging that this will not be always exclusively possible.

With the growing SJTREM reputation and workload it has become even more prudent to properly ensure the integrity within the editorial board. Thus, we thus like to introduce and welcome three new Associate Editors, which will undertake manuscript handling and executive tasks of submitted manuscripts. In particular, this will reduce the possibility of conflicting interests in case of submission from any of the editors or members of the editorial board, as manuscript handling may be covered by a larger and more diverse group of editors. The new editors are professor Maaret Castren (Karolinska Institute, Stockholm, Sweden), dr. David Lockey (London HEMS and Frenchay Hospital, Brighton, UK) and dr. Stefano Di Bartolomeo (University of Udine, Udine, Italy). With this addition to the editorial team we have not only vested in diversity but also recruited some of the most experienced researchers in Europe when it comes to research in trauma, resuscitation and emergency medicine. Welcome onboard!

The editors wishes to emphasize that the SJTREM endorses the standards set by the Vancouver-group, also known as the International Committee of Medical Journal Editors (ICMJE; http://www.icmje.org) as well as the Committee on Publication Ethics (COPE; http://publicationethics.org). The latter provides a forum for editors of academic journals to discuss issues relating to the integrity of the work submitted to, or published in, their journals. Examples include conflicts of interest, falsification and fabrication of data, plagiarism, unethical experimentation, redundant publication and authorship disputes. COPE encourages its members to seek investigation into possible misconduct by universities, hospitals or other funders. Flowcharts on how to handle the more common publication misconduct problems are accessible to all on the website (COPE; http://publicationethics.org). COPE has an independent "ombudsman" to adjudicate disputes between COPE members or between them and the organisation. COPE also publishes a Code of Conduct for Editors who are members of the organisation and will investigate complaints against Editors, if raised.

The Editors of SJTREM will continue to focus on every aspect of submission, peer-review and publication in the Journal and we welcome any correspondence on any such issue from authors and readers of the Journal.

## Competing interests

All authors are editors of the *Scandinavian Journal of Trauma, Resuscitation and Emergency Medicine*.

KGR and HML are employed by the Norwegian Air Ambulance Foundation, which as an idealistic organization pays for the article processing charge (APC) for all papers accepted for publication in the Journal. Else, there are no other financial, or otherwise stated, conflicts with the authors.
